# *LDLR* rs688 TT Genotype and T Allele Are Associated with Increased Susceptibility to Coronary Artery Disease—A Case-Control Study

**DOI:** 10.3390/jcdd5020031

**Published:** 2018-05-29

**Authors:** Chandan K. Jha, Rashid Mir, Naina Khullar, Shaheena Banu, S. M. S. Chahal

**Affiliations:** 1 Department of Human Genetics, Punjabi University, Punjab 147002, India; chandujha58@gmail.com (C.K.J.); smschahal1@gmail.com (S.M.S.C.); 2 Department of Medical Lab Technology, Faculty of Applied Medical Sciences, University of Tabuk, Tabuk 71491, Saudi Arabia; 3 Department of Zoology, Mata Gujri College, Fatehgarh Sahib 140407, India; naina306@gmail.com; 4 Sri Jayadeva Institute of Cardiovascular science & Research and Karnataka Institute of Diabetology, Bangalore 560069, India; shanbanubanu@gmail.com

**Keywords:** CAD—coronary artery disease, *LDLR*—low-density lipoprotein receptor, *LDLR* rs688C/T, SNP—single-nucleotide polymorphism, allele specific PCR, OR—odds ratio, CI—confidence interval

## Abstract

Purpose: The low-density lipoprotein receptor is responsible for the binding and uptake of plasma LDL particles and plays a critical role in maintaining cellular cholesterol homeostasis. *LDLR* gene SNP rs688 has been reported to be associated with increased plasma total and LDL cholesterol in several populations and can lead to elevated plasma LDL levels, resulting in an increased risk for atherosclerosis and coronary artery disease. This study aimed to explore genetic LDLR variant rs688 for its potential roles in coronary artery disease. Methodology: This study recruited 200 coronary artery disease patients and 200 healthy individuals. Genotyping of LDLR-rs688C > T gene variations was performed using the allele specific PCR method. Correlation of LDLR-rs688C > T gene variants with different clinicopathological features of coronary artery disease patients was performed. Pooled odds ratios (ORs) and 95% confidence intervals (CIs) were applied to evaluate the correlation of this microRNA polymorphism with coronary artery disease risk. Results: A significant difference was observed in genotype distribution among the coronary artery disease and matched healthy controls (*p* = 0.003). The frequencies of all three genotypes CC, CT, TT reported in the patient samples were 14%, 65% and 21% and in the healthy controls samples were 18%, 73% and 9%, respectively. The increased risk of developing CAD in Indian patients was found to be associated with *LDLR* rs688 TT genotype (OR = 3.0, 95% CI, 1.43 × 6.2; *p* = 0.003) RR 1.87 (1.20–2.91) *p* = 0.0037) and also the increased risk of developing CAD was reported to be associated with *LDLR* rs688 T allele (OR = 0.74, 95% CI, 1.57–0.97; *p* = 0.03) RR 0.85 (0.73–0.99) *p* = 0.03) compared to the C allele. Therefore, it was observed that more than a 3.0- and 0.74-fold increase risk of developing CAD was associated with TT genotype and T allele in Indian coronary artery disease patients. Conclusion: The findings indicated that *LDLR* rs688 TT genotype and T allele are associated with an increased susceptibility to coronary artery disease patients. LDLR-rs688C > T gene variation can be used as a predisposing genetic marker for coronary artery disease. Further studies with larger sample sizes are necessary to confirm our findings.

## 1. Introduction

Coronary Artery disease (CAD) is the most common type of heart disease which leads to morbidity and mortality among men and women, irrespective of race and ethnicity [1,2]. CAD is an intricate disease that is influenced by many environmental and familial risk factors [3,4]. In fact, many of the novel and traditional risk factors, such as lipoprotein-cholesterol, elevated low density lipoprotein, cholesterol, high blood pressure, smoking, obesity and diabetes are inadequate to fully understand who is at risk for CAD [5]. A complex series of events involving multiple biological pathways and genes are responsible for the progression of CAD [6,7]. It has been suggested by epidemiologic and genetic studies that certain genetic variants, which include polymorphisms in several genes, are associated with an inflated prevalence of CAD among high or low risk subjects. Study of the genetic elements would improve risk evaluation and offer better measures for the prevention and treatment of the CAD disease.

LDLR, the low-density lipoprotein receptor, is a cell surface glycoprotein, which has the ability of binding and up taking of plasma LDL particles and plays a significant role in maintaining the cellular cholesterol homeostasis [8]. Mutations in the *LDLR* gene can lead to a significant rise in plasma LDL levels, which in turn may result in an increased risk for atherosclerosis and coronary heart disease [9]. Various mutations of the *LDLR* have been described so far, influencing exons, splicing sites and the promoter regions. Mutations in genes of other proteins involved in LDL receptor adaptor protein (LDLRAP1) and LDL uptake and metabolism (ApoB) and in LDLR intracellular recycling (propoprotein convertase subtilisin/kexin type 9 serine protease, PCSK9) have also been shown to be involved in genetic hypercholesterolemia [10]. A single nucleotide polymorphism (SNP) in *LDLR* exon 12, rs688 is linked with low-density lipoprotein cholesterol (LDL-C) and coronary heart disease (CAD) in a gender-independent way [11,12].

*LDLR* rs688 acts as a modulator of alterative exon splicing, which can lead to a shift in the reading frame and an altered gene transcript [13,14]. The frequency of genetic polymorphism of LDLR (rs688) across the globe is summarized in Table 1. The non-coding SNPs in *LDLR* have also been found to have functions; for instance, rs17248720 in the intergenic region [15] and in the promoter region c.-139C > G [16], c.-101T > C, c.-121T > C [17], and c.-49C > T [18], are involved in regulation of gene expression and have been reported to cause familial hypercholesterolemia (FH). [19].

Notably, the allele frequencies and association analyses can differ widely amongst African, Americans, European Caucasians, Hispanics, Asians, and other ethnic groups. It is also noteworthy that associations found in one ethnic group may not translate to the same associations in other ethnic groups. The association of the single nucleotide polymorphisms with the severity of coronary arteries has been demonstrated [20,21]. Therefore, this study was pursued to examine the frequency of LDLR (rs688C > T) gene polymorphism in coronary heart diseases in an Indian population.

## 2. Materials and Methods

This study was a hospital-based case control study. Subjects were collected from different hospitals. Informed written consent was obtained for all study subjects. The research study was approved by the Ethical Committee of Punjabi University.

### 2.1. Inclusion Criteria

The case control study included clinically-confirmed coronary artery disease cases. The diagnosis was confirmed using electrocardiogram (ECG), echocardiogram stress test, cardiac catheterization and angiogram heart scan. Out of 200 CAD patients, 180 were males and 20 were females.

### 2.2. Exclusion Criteria

The exclusion criteria involved patients with who had previously had coronary bypass surgery or percutaneous transluminal coronary angioplasty (PTCA) because of their treated coronary status.

### 2.3. Collection of Clinical History

Informed written consent was obtained from all CAD patients, as well as healthy controls. Both CAD patients as well as healthy controls were interviewed using a structured questionnaire regarding epidemiological/demographic data, past history, history of addiction, particularly smoking, family history of MI or CAD. The detailed laboratory and clinical data were collected to determine relevant clinical history.

### 2.4. Coronary Artery Disease and Its Risk Factors

The traditional risk factors for coronary artery disease are high LDL cholesterol, low HDL cholesterol, high blood pressure, family history of a patient , diabetes, smoking, being post-menopausal for women and being older than 45 for men. The family history was the presence of a first degree relative with coronary artery disease at the age of <55 years for men and <60 years for women.

### 2.5. Coronary Angiography

Coronary angiography is a procedure that uses contrast dye, usually containing iodine, and X-ray pictures to detect blockages in the coronary arteries that are caused by plaque buildup. All 200 CAR cases underwent coronary angiography. This procedure is used to diagnose coronary heart disease and coronary microvascular disease after chest pain, sudden cardiac arrest, or abnormal results from tests, such as an electrocardiogram of the heart or an exercise stress test. It is important to detect blockages because, over time, they can cause chest pain, especially with physical activity or stress, or a heart attack. Coronary angiography detects the severity of coronary artery disease that can be expressed by the number of affected vessels (one, two, or three vessel disease).

### 2.6. DNA Extraction

DNA was extracted by using DNeasy Blood Kit (cat 69506) from Qiagen (Hilden, Germany) as per the manufactures instructions. The extracted DNA was dissolved in nuclease-free water and stored at 4 °C until use. The quality and integrity of DNA was checked using NanoDrop™ (Thermo Fisher Scientific 168 Third Avenue, Waltham, MA, USA).

### 2.7. LDLR (rs688) Genotyping

LDLR-rs688C > T gene polymorphism was detected using an allele-specific PCR. Allele-specific PCR is based on the use of sequence-specific PCR primers that allow amplification of test DNA, only when the target allele is contained within the sample. The LDLR-rs688C/T genotyping primers were designed using primer3 software [22], as depicted in Table 1.

AS-PCR was performed in a reaction volume of 25 μL containing template DNA (50 ng), F1 −0.35 μL, F2 −0.35 μL, RI −0.30 μL, and 25 pmol of each primer, and 10 μL of Green Master Mix (Qiagen). A final volume of 25 μL was adjusted by adding nuclease free ddH_2_O. The PCR cocktail was prepared as depicted in Table 2. Finally 2 μL of DNA in each PCR tube from each patient as well as control.

**Figure 1:**
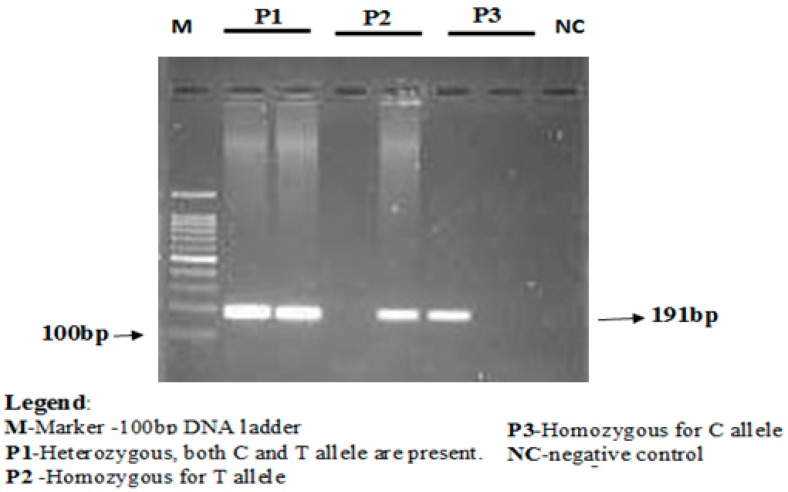
Genotyping of the *LDLR* rs688 (C/T) polymorphism by an allele-specific PCR assay. A 191-bp DNA fragment indicated the presence of the allele, while amplification failure indicated the bsence of the allele in the sample. In a 2% *w*/*v* agarose gel, L indicates molecular size standard, C and T indicate the product from reaction C and reaction T, respectively, amplifiled from selected DNA sample of each possible genotypes (CC, CT and TT) and H_2_O (negative control).

The concentration of the mutant-specific primer was raised, and concentrations of common reverse primer were lowered to favor amplification from the mutant allele. The annealing temperature was lowered from 67 to 68 °C to favor the binding of both forward primers that contained mismatches with the templates. PCR conditions used were as follows, initial denaturation for 3 min at 94 °C followed by 35 cycles of 30 s at 94 °C (denaturation), 30 s at 67 °C (annealing), 1 min at 72 °C (elongation), and finally followed by 10 min at 72 °C (final elongation). PCR products were stored at 4 °C till further analyses. The amplification products were separated using electrophoresis via 2% agarose gel stained with ethidium bromide. Lengths of PCR products formed were 191 bp for both rs688C allele and rs688T allele, as depicted in Figure 1.

The number of cycles was increased from 30 to 40 cycles, significantly enhancing the yields of all three PCR products. Together, these changes resulted in a more robust amplification of the mutant allele and a less competing reaction from the control, as evidenced by the relative intensities of the corresponding bands on agarose gel electrophoresis.

### 2.8. Statistical Analysis

Deviations from Hardy-Weinberg disequilibrium (HWD) were calculated using the Chi-square (χ^2^) goodness-of-fit test. Group differences were compared using Student’s two-sample *t*-test or one-way analysis of variance (ANOVA) for continuous variables and Chi-squared for categorical variables. Differences of the LDLR-rs688C/T allele and genotype frequencies between groups were evaluated using the Chi-square test. The associations between LDLR-rs688C/T genotypes and risk of CVD were estimated by computing the odds ratios (ORs), risk ratios (RRs) and risk differences (RDs) with 95% confidence intervals (CIs). Allele frequencies among cases, as well as controls, were evaluated using the Chi-square Hardy-Weinberg equilibrium test. A *p* value < 0.05 was considered significant. All statistical analyses were performed using SPSS 16.0 (IBM, Chicago, IL, USA)

## 3. Results

### 3.1. The Hardy-Weinberg Equilibrium Analysis

The genotype distributions and allele frequencies of the SNPs located in the LDLR-rs688C/T showed that no deviations were detected in HWE (all *p* > 0.05) (χ^2^ = 0.44 *p* ≤ 0.612) in the patient group. Similarly, the genotype distributions and allele frequencies of the LDLR-rs688C/T showed that no deviations were detected in HWE (all *p* > 0.05) (χ^2^ = 0.52 *p* ≤ 0.712) in the control. Thus, we chose 10% of the samples from the normal control group, randomly, to review genotyping results, showing that the accuracy rate was more than 99%.

### 3.2. Study Population

All demographic features of the subjects are shown in Table 3. This population-based case–control study was done with 200 clinically-confirmed CAD patients and 200 age-matched healthy controls with no history of any types of diseases and was not related to the patients. In brief, the total number of CAD patients and the same number of matched healthy control were analyzed. The research study was conducted at the Department of Human Genetics, Punjabi University, Patiala-147002, India. The research study was approved by the Ethical Committee of Punjabi University and written informed consent was obtained from all subjects before enrollment. The research complied with the principles of the Declaration of Helsinki.

### 3.3. DNA Extraction

Blood samples were collected from participants in EDTA-containing tubes. Of the 200 consecutive CAD patients, 180 (90%) were males and 20 (10%) were females, whereas, among the healthy controls, 176 (88%) were males and 24 (12%) were females. Subjects were classified on the basis of age, among the CAD patients, 90 (45%) were below or equal to 50 years age and 110 (55%) were above 50. The correlation between LDLR polymorphism and CAD patients is depicted in Table 4.

### 3.4. Case-Control Genotype Distribution

A significant difference was observed in genotype distributions among the CAD cases and matched healthy controls (*p* = 0.0031). The frequencies of all three genotypes CC, CT, TT reported in patients were 14%, 65% and 21%, and in healthy controls, 18%, 73% and 9%, respectively. This study observed that a high percentage of the TT (21%) genotype was found in patients compared to control TT (9%) genotype, as depicted in Table 5.

### 3.5. Risk of CAD with LDLR rs688C > T gene Polymorphism in CAD Patients

A multivariate analysis based on logistic regression, such as odds ratio, risk ratio and risk difference with 95% confidence intervals, were calculated for each group to estimate the association between the *LDLR* rs688C > T variant and risk of CAD in Indian patients. The odds ratio and risk ratio, with a 95% confidence interval, was calculated for each group to estimate the degree of association between the *LDLR* rs688 C > T variant and risk of CAD risk in Indian patients, depicted in Table 6. The increased risk of developing CAD in Indian patients was found to be associated with *LDLR* rs688 TT genotype (OR = 3.0, 95% CI, 1.43–6.2; *p* = 0.0037) RR 1.87 (1.20–2.91) *p* = 0.0037) and also an increased risk of developing CAD was reported to be associated with *LDLR* rs688 T allele (OR = 0.74, 95% CI, 1.57–0.97; *p* = 0.03) RR 0.85 (0.73–0.99) *p* = 0.03). Therefore, it was observed that more than a 3.0- and 0.74-fold increase risk in developing CAD was associated with TT genotypes and the T allele in Indian patients.

## 4. Discussion

In India, more than 10.5 million deaths occur annually, and it has been reported that CAD led deaths occurred in 20% in men and 16.9% in women. According to 2010–2013 RGI data (27), proportionate mortality from CAD increased to 23% of total and 32% of adult deaths in the years 2010–2013 [23]. Coronary artery disease (CAD) is a leading cause of morbidity and mortality worldwide and has become a major public health burden in India [24]. 

Traditional risk factors, such as lipid-rich diet, advanced age, smoking, hypertension, diabetes mellitus and dyslipidemia, are associated with an increased risk of CAD. RNA made containing the rs688 (T) SNP, a variant near exon 12 of the low-density lipoprotein receptor, which is a receptor for ApOE proteins, is spliced at a lower efficiency in males. Recent genome-wide association studies have highlighted that common variations at the *LDLR* locus are strongly associated with proatherogenic lipid profile and with CAD [25]. 

In our study, we reported a significant difference in genotype distribution among the CAD cases and matched healthy controls (*p* = 0.0031). The frequencies of all three genotypes, CC, CT, and TT, reported in patients were 14%, 65% and 21% and in healthy controls 18%, 73% and 9% respectively. The *LDLR TT* genotype as well as LDLR T allele was associated with increased susceptibility to CAD in Indian patients. The frequencies of LDLR rs688 genotypes CC, CT, and TT were obtained from different studies performed in different countries, as shown in Table 7. Teslovich et al. [26], through genome-wide association studies reported single nucleotide polymorphisms at the *LDLR* locus that contribute to inter-individual variation in serum lipid concentrations. In the same study it was reported that several SNPs within *LDLR*, such as rs12983082, rs2738446, rs1799898, rs9789302, and rs5925, were in linkage disequilibrium with *LDLR* rs688C/T *r*^2^ > 0.8, but none were associated with plasma lipids, suggesting that rs688 is the causative underlying polymorphism. Our study observed that a higher percentage of TT (21%) genotype was found in patients than in healthy control TT (9%). Similar results were in obtained in Italy (21%) and the USA (21%), whereas a lesser percentage of the TT genotype (2% and 4%) was reported in two studies from Taiwan, as depicted in Table 7.

It was reported that the minor variant of LDLR rs688 (Asn^591^ ACC→ACT) gene has been reported to be associated with a 4–10% increase in plasma cholesterol levels in several independent populations [29]. Mutations in the *LDLR* gene can lead to elevated plasma LDL levels, resulting in an increased risk for atherosclerosis and coronary heart disease [30]. Several studies have established that the rs688 SNP is associated with significantly decreased LDLR exon 12 splicing efficiency in women in vivo. The rs688 C/T in exon 12 has been recently shown to alter splicing efficiency, with the T allele being associated with increased total and LDL-cholesterol levels in premenopausal women. These effects on splicing may be physiologically relevant because of the presence of rs688 minor allele being associated with increased total and LDL-cholesterol in female members of the Framingham Offspring Study. It was reported that an LDLR SNP present in approximately 60% of Caucasians is associated with significant (10%) increases in total and LDL-cholesterol in pre-menopausal women [31]. There is clear epidemiological evidence that increased levels of LDL lead to cardiovascular disease (mostly coronary disease), and it is estimated that elevated cholesterol contributes to 4.4 million deaths per year worldwide [32]. Samani et al. [33], reported that the minor allele at the LDLR rs688 SNP has recently been associated with increased risk of coronary artery disease in a combined genome-wide analysis of British and German cohorts. Similar results were documented in our study, in which LDLR rs688 SNP is associated with an increased risk of developing coronary artery disease.

## 5. Conclusions

The findings indicated that the *LDLR* rs688 TT genotype and the T allele are associated with increased susceptibility to coronary artery disease in patients in an Indian population. *LDLR* rs688 can be used as a predisposing genetic marker for coronary artery disease. Further studies with larger sample sizes are necessary to confirm our findings.

## Figures and Tables

**Table 1:** Allele specific PCR primers for LDLR-rs688C/T gene polymorphism.

		Primer Sequence	Product Size
Forward F1	C allele	5′-CACTCCATCTCAAGCATCGATGTCAAC-3′	191 bp
Forward F2	T allele	5′-CACTCCATCTCAAGCATCGATGTCAAT-3′	
Reverse		5′-CAACCAGTTTTCTGCGTTCATCTTG-3′	
Amino acid change		N [Asn591] ⇒ N [Asn591]	
Nucleotide change		C/T (FWD)	

**Table 2:** Preparation of PCR cocktail for LDLR-rs688C > T polymorphism.

Reagent	1×
PCR master mix	10 μL
Forward primer F1	0.35 μL
Forward primer F2	0.35 μL
Reverse primer R	0.30 μL
Nuclease free water	12 μL
Total volume	23 μL
Finally add	2 μL DNA
Total volume	25 μL

**Table 3:** Baseline characteristics of CAD patients and controls.

Variables	CAD Cases	Healthy Controls
	200	200
Gender difference		
Males	180 (90%)	176 (88%)
Females	20 (10%)	24 (12%)
Age difference		
Age ≤ 50	90 (45%)	88 (44%)
Age > 50	110 (55%)	112 (56%)
	**No of CAD Cases**	%
Cholesterol		
≤200 mg	176	(88%)
>200 mg	24	(12%)
RBS		
RBS ≤ 140 mg	129	(64.5%)
RBS > 140 mg	71	(35.5%)
HDL		
≤40 mg	166	(83%)
>40 mg	34	(17%)
LDL		
≤100 mg	150	(75%)
>100 mg	50	(25%)
TGL		
≤150 mg	105	(52.5%)
>150 mg	95	(47.5%)
*Coronary heart disease* (*CHD*)		
Yes	23	11.5%
No	177	88.5%
Hypertension		
Yes	53	26.5%
No	147	73.5%
Type 2 Diabetes		
Yes	54	27%
No	146	73%
Smoking		
Yes	121	60.5%
No	79	39.5%
Alcohol		
Yes	71	35.5%
No	129	64.5%
Pan Masala		
Yes	4	2%
No	196	98%

**Table 4:** Allele and genotype frequency of *LDLR* gene polymorphism of study cohorts.

Subjects	N=	C/C	C/T	T/T	DF	X^2^	*p* Value
Correlation with gender							
Males	180	25	117	38	0.03	2	0.98
Females	20	03	13	04			
Correlation with Age							
Age ≤ 50	90	12	64	14	3.3	2	0.19
Age > 50	110	16	66	28			
Correlation with RBS							
RBS ≤ 140 mg	129	17	86	26	0.45	2	0.79
RBS > 140 mg	71	11	44	16			
Correlation with Cholesterol							
Cholesterol ≤ 200 mg	176	25	112	39	1.4	2	0.49
Cholesterol > 200 mg	24	03	18	03			
Correlation with HDL							
HDL ≤ 40 mg	166	25	104	37	2.39	2	0.30
HDL > 40 mg	34	03	26	05			
Correlation with LDL							
LDL ≤ 100 mg	150	20	97	33	0.49	2	0.78
LDL > 100 mg	50	08	33	09			
Correlation with TGL							
TGL ≤ 150 mg	105	16	66	23	0.48	2	0.78
TGL > 150 mg	95	12	64	19			
Correlation with hypertension							
Hypertension	53	10	32	11	1.46	2	0.48
No hypertension	147	18	98	31			
Correlation with Diabetes							
Diabetes	54	07	34	13	0.44	2	0.80
No Diabetes	146	21	96	29			
Correlation with CHD							
CHD	23	07	11	05	6.2	2	0.04
No CHD	177	21	119	37			
Correlation with smoking							
Smoking	121	16	84	21	2.99	2	0.22
No Smoking	79	12	46	21			
Correlation with alcohol							
Alcohol	71	09	46	16	0.26	2	0.87
No Alcohol	129	19	84	26			

**Table 5:** Genotype frequency of *LDLR* rs688C > T polymorphism of study cohorts.

Allele/Genotype	C/C	C/T	T/T	Chi-Square	*df*	*p* Value
CAD patients	28 (14%)	130 (65%)	42 (21%)	11.53	2	0.0031
Controls	36 (18%)	146 (73%)	18 (9%)			

**Table 6:** Association of *LDLR* rs688C > T gene variation with CAD.

Genotypes	Healthy Controls		CAD Cases		OR (95% CI)	Risk Ratio (RR)	*p*-Value
	(*n* = 200)	%	(*n* = 200)	%			
Codominant model							
LDLR-CC	36		28		Ref	Ref	
LDLR-CT	146		130		1.14 (0.66–1.97)	1.06 (0.883–1.35)	0.62
LDLR-TT	18		42		3.0 (1.43–6.2)	1.87 (1.20–2.91)	0.0037
Dominant model							
LDLR-CC	36		28		Ref	Ref	
LDLR-(CT + TT)	164		172		1.34 (0.78–2.30)	1.15 (0.90–1.46)	0.250
Recessive model							
LDLR-(CC + CT)	182		312		Ref	Ref	
LDLR-TT	18		42		1.36 (0.76–2.43)	1.22 (0.82–1.83)	0.310
Allele							
LDLR-C	218		340		Ref	Ref	
LDLR-T	182		214		0.74 (0.57–0.96)	0.85 (0.73–0.98)	0.032

**Table 7:** Frequency of *LDLR* rs688C/T in different countries.

Country	Subjects	N=	CC	%	CT	%	TT	%	
Taiwan	Cases	447	295	66	143	32	9	2	[27]
	controls	430	292	68	121	28	17	4	
United States	Cases	84	21	25	45	53.6	18	21.4	[28]
	controls	69	29	42	30	43.5	10	14.5	
Iran	Cases	170	55	32.35	79	46.47	36	21.18	[20]
	controls	104	41	39.81	48	45.63	15	14.56	
Taiwan	Cases	815	538	66	251	30.8	26	3.2	[26]
	controls	430	295	68.6	118	27.45	17	3.95	
Italy	Cases	692	208	30.1	335	48.4	149	21.5	[12]
	controls	291	109	37.5	123	42.3	59	20.2	
India	Cases	200	28	14	130	65	42	21	
	controls	200	36	18	146	73	18	9	
